# Supplementary material for: A diamond-bearing core-mantle boundary on Mercury
Source: Nat Commun. 2024 Jun 14;15:5061. doi: 10.1038/s41467-024-49305-x (PMC11178936; doi:10.1038/s41467-024-49305-x)
Supplement: Supplementary file 1 — Supplementary Information [file 41467_2024_49305_MOESM1_ESM.pdf]

Supplementary Information for

A diamond-bearing core-mantle boundary on Mercury

Yongjiang Xu[1], Yanhao Lin[1,*], Peiyan Wu[1,2], Olivier Namur[3], Yishen Zhang[3] & Bernard Charlier[4]

[1]Center for High Pressure Science and Technology Advanced Research, Beijing 100193, People's Republic of China

[2]School of Earth Sciences and Resources, China University of Geosciences, Beijing 100083, People's Republic of China

[3]Earth and Environmental Sciences, KU Leuven, 3001 Leuven, Belgium

[4]Department of Geology, University of Liege, 4000 Sart Tilman, Belgium

*Corresponding author. Email: yanhao.lin@hpstar.ac.cn (Y.L.)

Contents

Supplementary Table S1

Supplementary Figure S1

Supplementary Figure S2

Supplementary Figure S3

Supplementary Figure S4

Supplementary Figure S5

Supplementary Figure S6

Supplementary Figure S7

Supplementary Figure S8

Supplementary Figure S9

Supplementary Figure S10

Supplementary Figure S11

	EH-EL (BSM)	Mer8	Mer15	$SiCore_4$	$SiCore_8$
SiO_2	61.21	54.82	47.91	58.47	55.27
TiO_2	0.12	0.14	0.16	0.12	0.14
Al_2O_3	3.23	3.70	4.26	3.45	3.71
Cr_2O_3	0.45	0.52	0.60		
MnO	0.25	0.28	0.33		
MgO	32.64	37.32	43.04	35.01	37.72
CaO	1.48	1.62	1.87	1.52	1.65
Na_2O	1.21	1.37	1.58	1.29	1.39
K_2O	0.11	0.13	0.15	0.13	0.13
P_2O_5	0.32	0.10	0.10		
Total	101.02	100.00	100.00	99.99	100.01

Supplementary Table S1 Average composition of the silicate fraction of EH-EL meteorites, starting compositions considering a bulk silicate Mercury compositionally similar to the silicate fraction of an enstatite chondrite with 8 or 15% Si partitioned into Mercury's core (compositions Mer8 and Mer15), and Potential S-free magma ocean compositions for Mercury ($SiCore_4$, $SiCore_8$). All data in wt%.

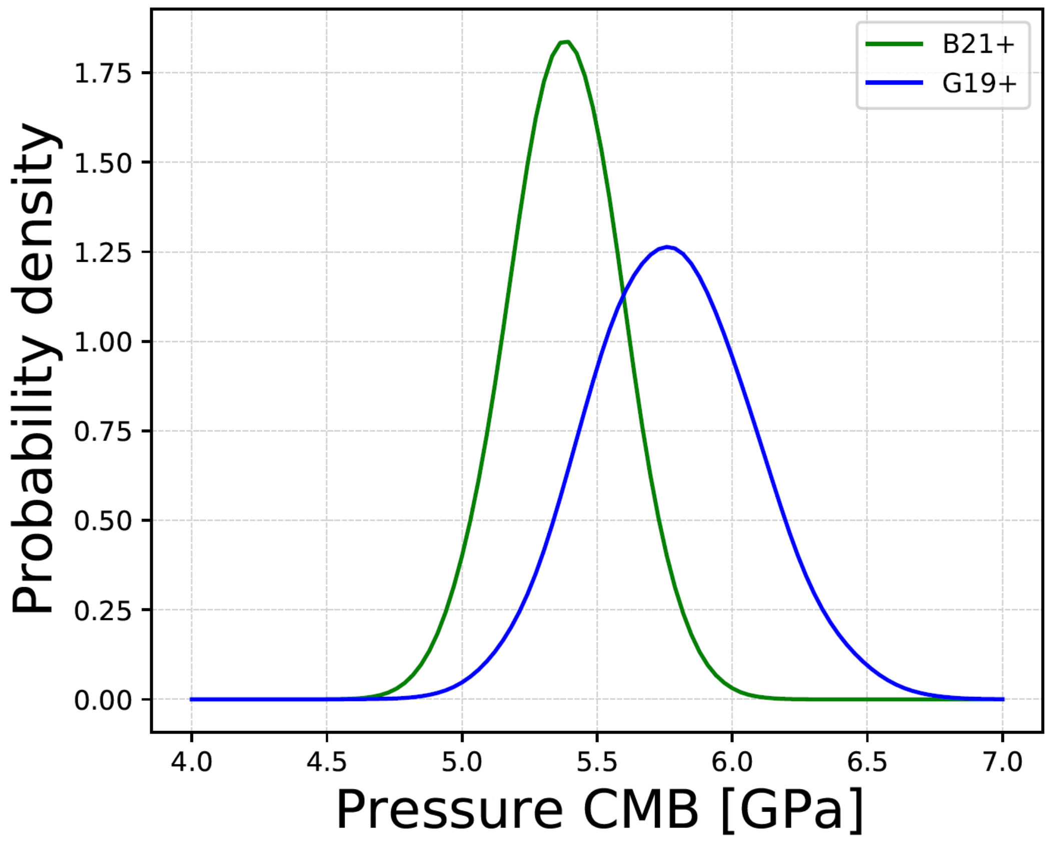

Supplementary Figure S1 Calculated pressure at Mercury's CMB with the internal structural modeling results of Goossens et al. (2022) using the normalized polar moment of inertia of Genova et al. (2019) (blue line) and that of Bertone et al. (2021) (green line).

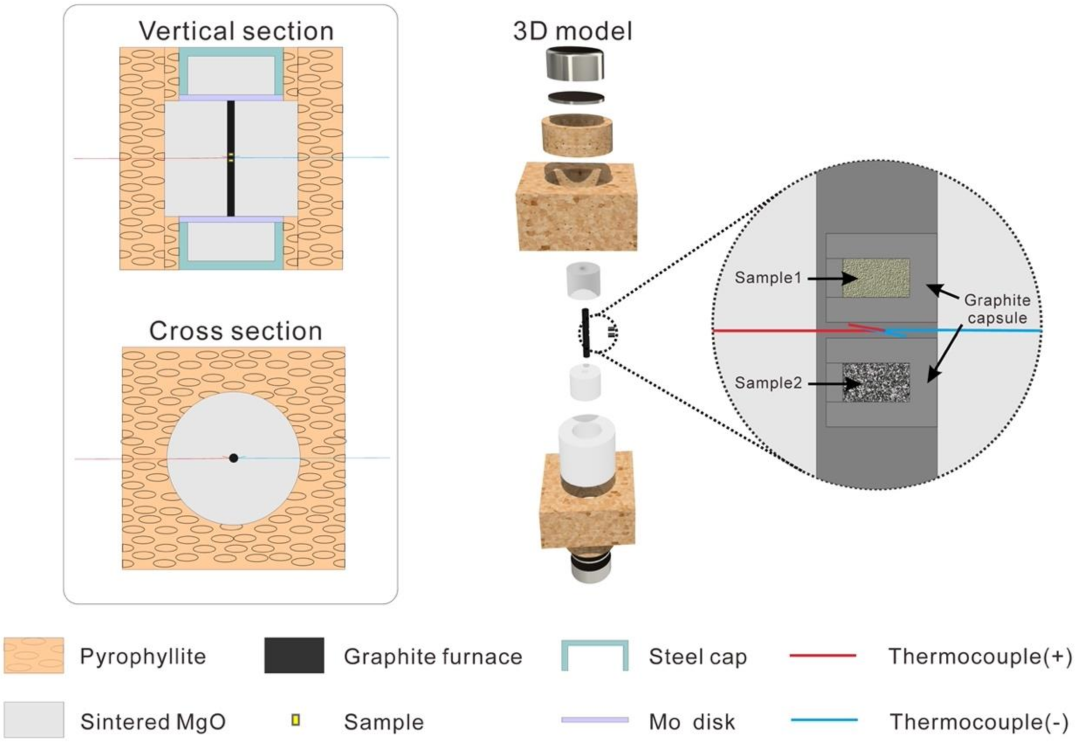

Supplementary Figure S2 Experimental assembly shown in vertical section, cross section, and 3D model. A slim graphite rod (2.5 mm in diameter) is placed vertically in the center of the assembly, and sample capsules are inserted into this rod. In addition, two sample capsules can be done at the same time, and the thermocouple junction is placed between the capsules in the center of the assembly. The length of cube edge is 38.5 mm. This figure is modified after the ref. 87.

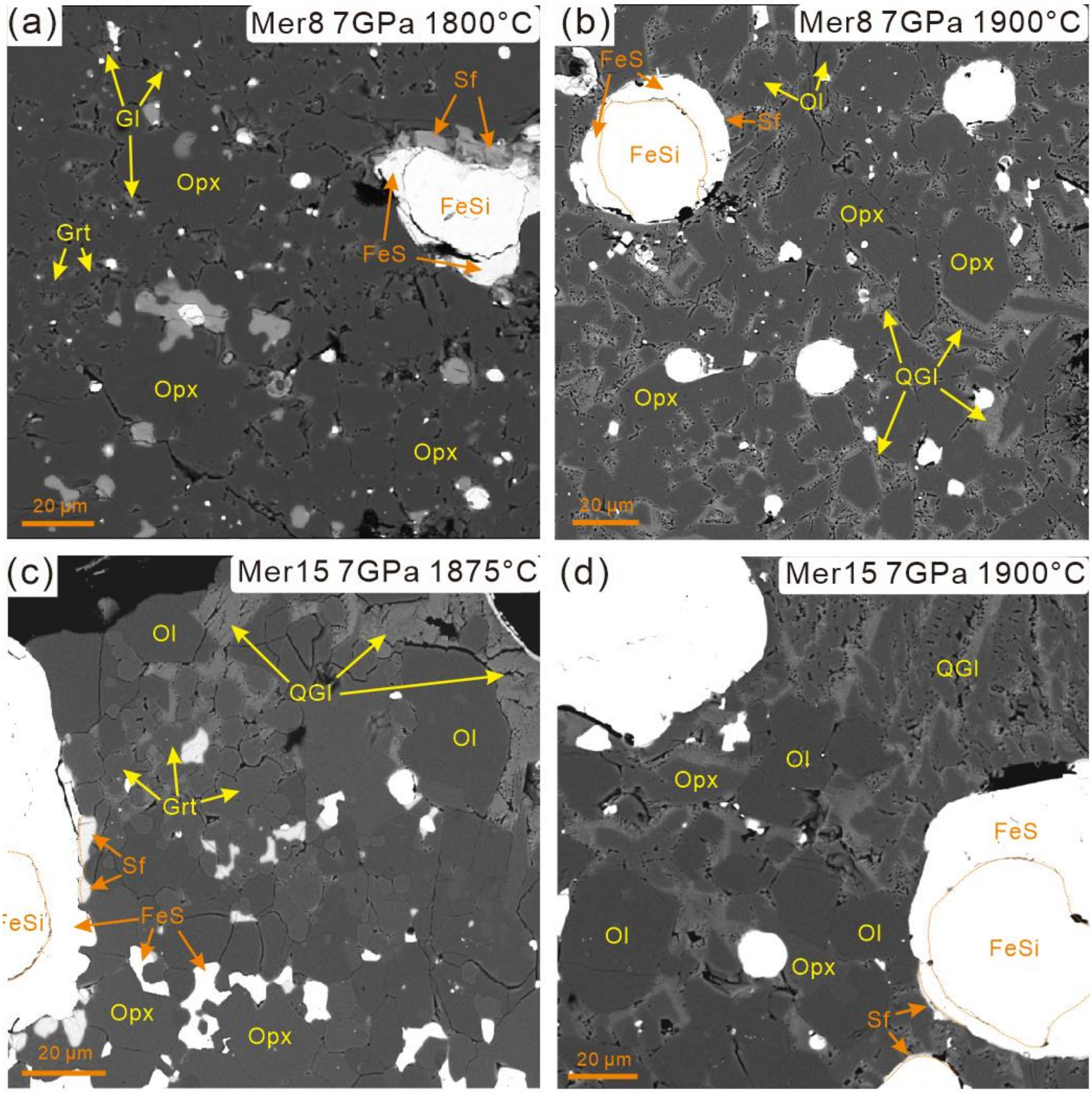

Supplementary Figure S3 Representative backscattered electron (BSE) images of experimental products at 7 GPa for the starting compositions Mer8 and Mer15 (see Table S1). The silicate mineral phases in the panels a–b are Opx+Grt (a), Ol+Opx (b), Ol+Opx+Grt (c), and Ol+Opx (d), respectively. The analyzed chemical compositions of each phase are shown in the Supplementary Data 1. Experimental conditions are listed at the top of each panel. QGl, glass with quenchable texture; Gl, glass; Ol, olivine; Opx, orthopyroxene; Grt, garnet; Sf, sulfide [(Mg,Fe,Ca)S].

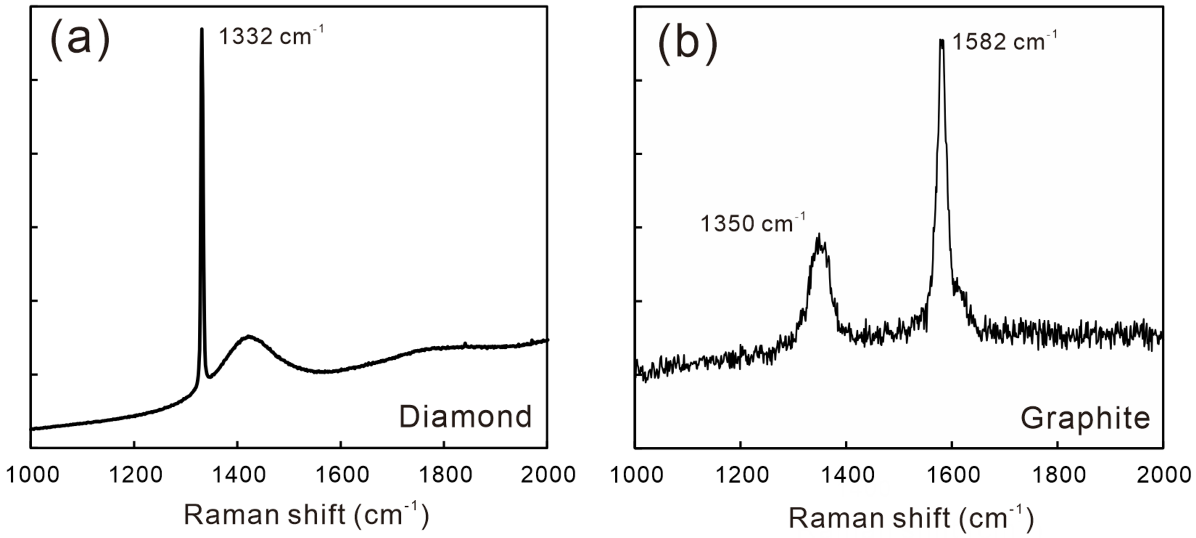

Supplementary Figure S4 Representative Raman spectra of “graphite/diamond” capsules of our experimental products. The experimental temperatures of panels (a) and (b) are 2323 and 2373 K, respectively.

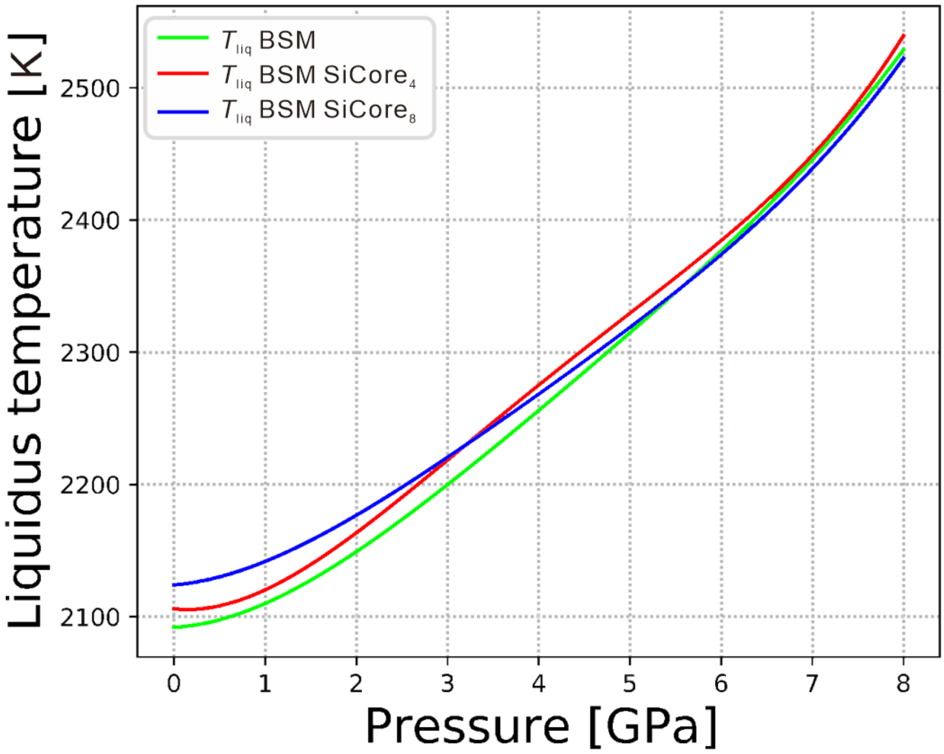

Supplementary Figure S5 Calculated liquidus curves for three potential bulk compositions of Mercury's magma ocean (see Table S1).

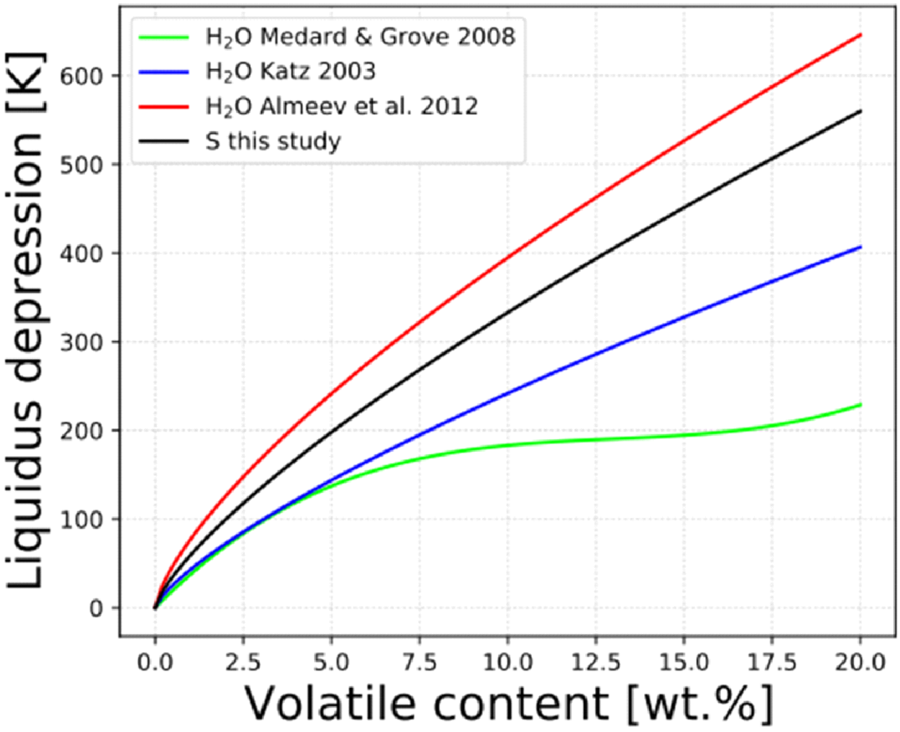

Supplementary Figure S6 Calculated liquidus depression as a function of the content of the dissolved volatile species (H_2O or S).

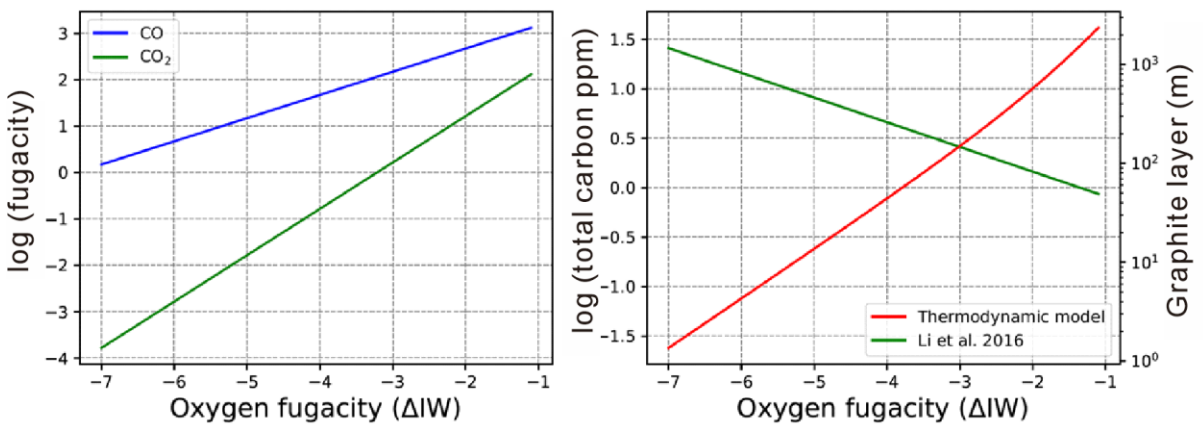

Supplementary Figure S7 Left: calculated fugacities of CO and CO_2 as a function of fO_2 (ΔIW) using Eqs. 18a and 18b. Right: calculated carbon content and thickness of the graphite flotation layer using Eqs. 19 (red) and 17 (green).

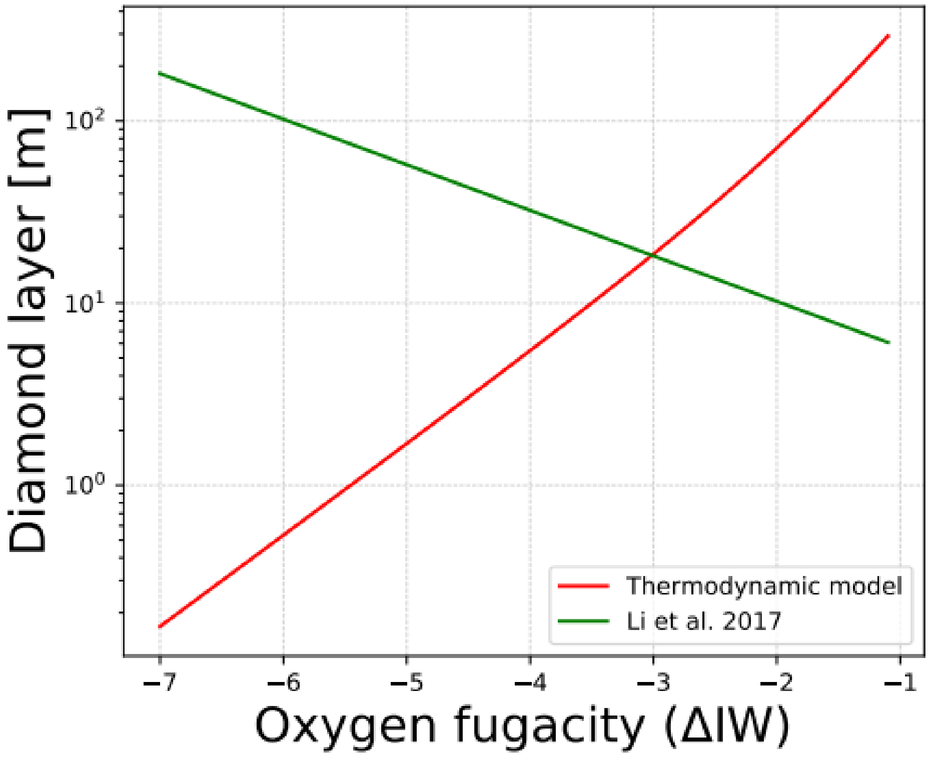

Supplementary Figure S8 Calculated thickness of a diamond layer crystallized from the magma ocean.

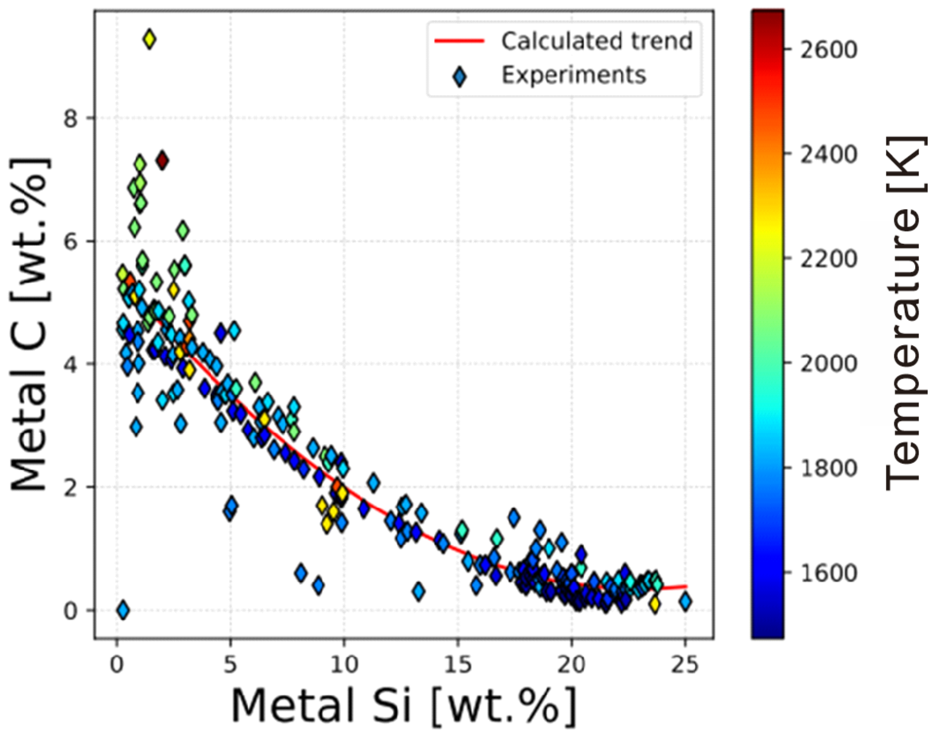

Supplementary Figure S9 Carbon content in carbon-saturated FeSi metal.

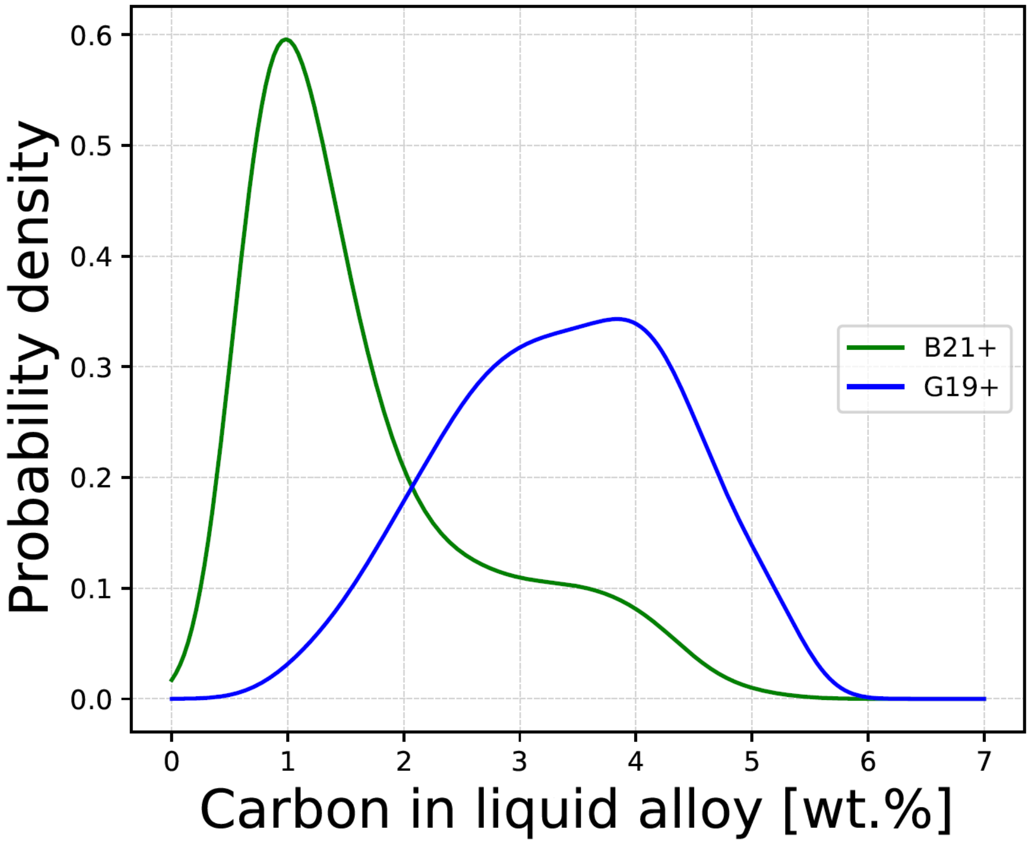

Supplementary Figure S10 Calculated C content of Mercury's bulk core based on Si-C correlation in molten metallic melt and interior models from[25] with MOI = 0.333 ± 0.005[24] and MOI = 0.343 ± 0.006[26].

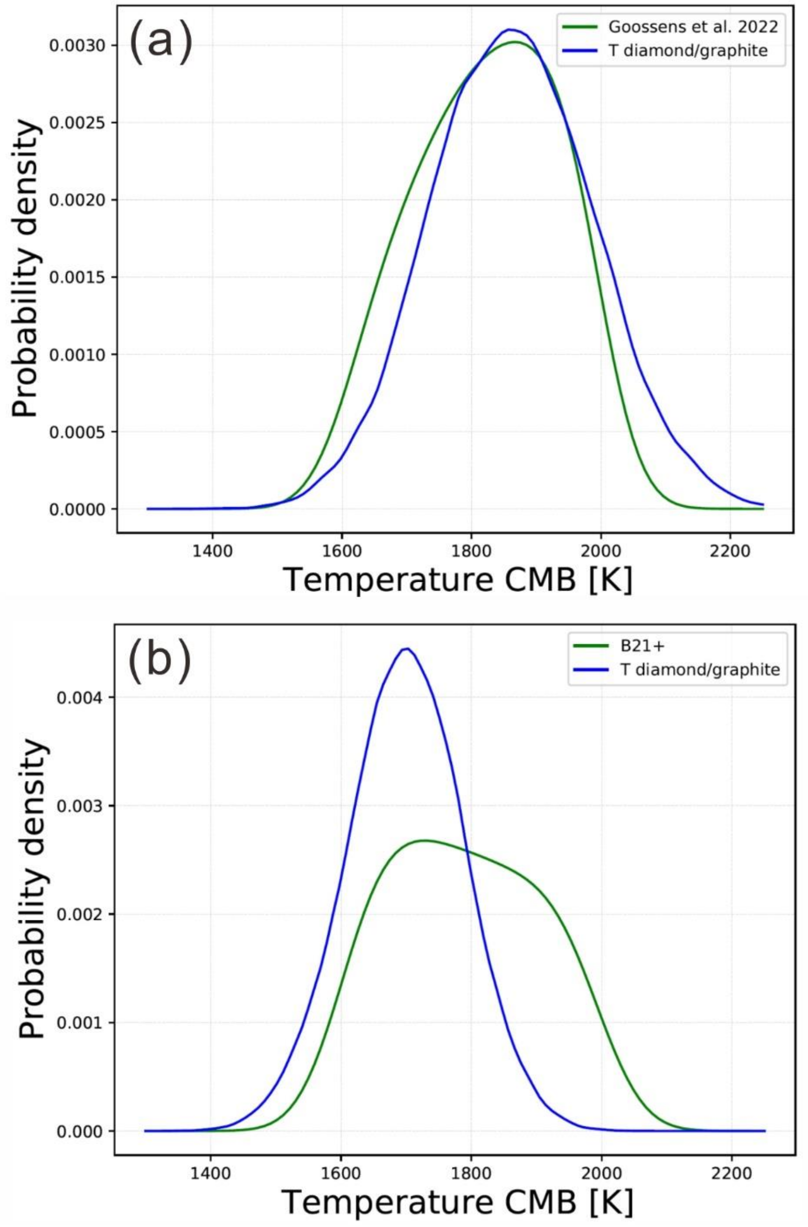

Supplementary Figure S11 Density distributions of the present core-mantle boundary temperature (green) and the graphite/diamond transition temperature at the present core-mantle boundary pressure (blue). Panel (a) shows interior models from Goossens et al. (2022) with MOI = 0.333 ± 0.005 (Genova et al., 2019); Panel (b) shows data for MOI = 0.343 ± 0.006 (Bertone et al., 2021).
