# Supplementary material for: A diamond-bearing core-mantle boundary on Mercury
Source: Nat Commun. 2024 Jun 14;15:5061. doi: 10.1038/s41467-024-49305-x (PMC11178936; doi:10.1038/s41467-024-49305-x)
Supplement: Supplementary file 3 — Description of Additional Supplementary Files [file 41467_2024_49305_MOESM3_ESM.pdf]

Description of Additional Supplementary Files

Supplementary Data 1: Summary of experimental conditions and results.
